# Prediction Model for Brain Metastasis in Patients With Metastatic Germ‐Cell Tumors

**DOI:** 10.1002/cam4.70649

**Published:** 2025-02-06

**Authors:** Tareq Salous, Ryan Ashkar, Sandra K. Althouse, Clint Cary, Timothy Masterson, Nasser H. Hanna, Jennifer King, Lawrence H. Einhorn, Nabil Adra

**Affiliations:** ^1^ Division of Hematology‐Oncology Indiana University Simon Comprehensive Cancer Center Indianapolis Indiana USA; ^2^ Department of Biostatistics and Health Data Science Indiana University Indianapolis Indiana USA; ^3^ Department of Urology Indiana University School of Medicine Indianapolis Indiana USA

**Keywords:** brain metastasis, germ cell tumors, prediction model, prognosis

## Abstract

**Background:**

Brain metastasis (BM) is an independent adverse prognostic factor in metastatic germ cell tumors (mGCT). We aimed to establish an effective and practical BM prediction model.

**Patients and Methods:**

Between January 1990 and September 2017, 2291 patients with mGCT who were treated at Indiana University were identified. Patients were divided into two categories: BM present (*N* = 154) and BM absent (*N* = 2137). Kaplan–Meier methods were used to analyze progression free survival (PFS) and overall survival (OS). Logistic regression was used to determine a predictive model for whether BM was present. The data was separated into training and validation datasets with equal numbers of events in each.

**Results:**

The 2‐year PFS and OS for patients with versus without BM: 17% versus 65% (*p* < 0.001) and 62% versus 91% (*p* < 0.001) respectively. Among the 154 patients with BM, 64 (42%) had radiation only (whole‐brain radiotherapy or gamma knife), 22 (14%) had BM‐surgery only, 14 (9%) had both radiation and BM‐surgery. 54 patients (35%) did not receive local therapy for BM. Stepwise selection was used to determine the best model with *p* < 0.15 as the entry and staying criteria. The model with the largest ROC AUC was used moving forward. The model was tested in the validation dataset. A model was generated including age at diagnosis ≥ 40, choriocarcinoma predominant histology, pre‐chemotherapy hCG≥ 5000, presence of pulmonary metastases size < 3, or ≥ 3 cm, and presence of bone metastasis. Patients with score of 0, 1, 2, 3, 4, 5, 6, 7, 8 points had a 0.6%, 1.4%, 3.5%, 8.2%, 18.3%, 36%, 58%, 78%, 90% probability of having BM, respectively.

**Conclusions:**

The prediction model developed in this study demonstrated discrimination capability of predicting BM occurrence in mGCT and can be used to identify high‐risk patients.

## 1 Introduction

Germ cell tumors (GCTs) are the most common cancer in young males between the ages of 15 and 35 [1]. While GCTs are highly curable, they can exhibit an aggressive behavior, including metastasis to various organs. The International Germ Cell Cancer Consensus Group (IGCCCG) defined a classification system based on identification of clinically independent prognostic features such as extent of disease and post orchiectomy levels of serum tumor markers. This classification system categorizes patients into good‐, intermediate‐, or poor‐risk groups [2]. Patients with good and intermediate risk disease have excellent prognosis and are mostly cured with 1st line chemotherapy. In contrast, about 50% of poor risk patients will progress after first‐line chemotherapy and require additional therapy [3].

Patients with non‐seminoma who present with non‐pulmonary visceral metastasis are classified as poor risk patients based on IGCCCG. One of the most concerning sites of metastasis is the brain; it can significantly impact a patient's prognosis and quality of life. Brain metastasis (BM) is relatively rare, but still occurs in about 2%–5% of patients with advanced GCTs [4, 5]. It is considered an independent prognostic factor that is associated with worse outcomes [6]. BM is more common with non‐seminoma histology, usually preceded by systemic disease [7], and can be present at time of initial presentation or at relapse.

Only a small proportion of patients have symptoms related to BM. In a study of 582 patients, half of the patients who had BM at time of initial presentation, and one third of patients who had BM at relapse were asymptomatic [6]. Existing guidelines are inconclusive regarding whether to screen for BM in high‐risk patients. Developing a predictive model for BM in GCTs is of paramount importance; as it can aid in early detection, personalized treatment planning, and potentially improve patient outcomes [8].

In this study, we aim to establish an effective and practical BM prediction model in patients with metastatic GCTs.

## 2 Methods

### 2.1 Patients

The prospectively maintained Indiana University database for testicular cancer was used to identify eligible patients. Patients who were ≥ 18‐year‐old and treated for metastatic GCTs at Indiana University between January of 1990 and September 2017 were included in the study.

### 2.2 Study Monitoring

The study used data from the prospectively maintained Indiana University GCT database. The database is regulated by an IRB approved protocol. A waiver for consent is obtained from the IRB through the GCT database protocol.

### 2.3 Statistical Analysis

Data analysis was conducted using SAS software version 9.4 (SAS Institute Inc., Cary, NC). Baseline demographic and disease characteristics were summarized as median (range) for continuous variables and number (percent) for categorical variables. The Kaplan–Meier method was used to analyze progression‐free survival (PFS) and overall survival (OS), with the log‐rank test used to compare groups. For PFS, patients who did not progress or die were censored at their last follow‐up visit. For OS, patients who did not die were censored at their last follow‐up visit. Medians with 95% confidence intervals were calculated, along with the 1‐, 2‐, and 5‐year probabilities. The Cox proportional hazards model was also used to compare OS in patients with BM based on time of presentation. Logistic regression using stepwise selection was used to determine a predictive model for the development of BM.

### 2.4 Model Construction

To construct BM prediction model, we utilized patient data from the Indiana University database, selecting a set of predictor variables that have been previously identified as clinically relevant for metastatic GCT. The model was constructed using logistic regression, a well‐established method for classification. Variables selected for inclusion in the model development included the primary site, histology (choriocarcinoma vs. non‐choriocarcinoma), age group (< 40 vs. ≥ 40), liver metastasis, bone metastasis, pulmonary metastasis size (none, < 3 cm, ≥ 3 cm), alpha‐fetoprotein (AFP) pre‐chemotherapy ≥ 1000, and human chorionic gonadotropin (hCG) pre‐chemotherapy ≥ 5000. Stepwise selection was used to refine the model, with criteria for variables entering or staying in the model if their *p*‐value was less than 0.15. The performance of the model was assessed using the receiver operating characteristic (ROC) area under the curve (AUC), which quantifies the model's discriminative ability. The model with the largest ROC AUC was used moving forward.

To validate the model, the data were randomly split into training (50%) and validation (50%) datasets. The training dataset (*N* = 1146 patients with 77 events) was used to build the model using stepwise selection, while the validation dataset (*N* = 1145 patients with 77 events) was used to test the model's predictive accuracy by confirming a reasonable ROC AUC. The beta values from the validation dataset, using the closest integer, were used to determine the points to be assigned for each condition. A total score was generated and run on the validation dataset to determine probability estimates for each possible total score value.

## 3 Results

2291 patients with metastatic GCT treated at Indiana University between January 1990 and September 2017 were identified. Patients were divided into two categories: BM present (*N* = 154) and BM absent (*N* = 2137). The baseline characteristics for the two groups are listed in Table 1.

**TABLE 1:** Patient and disease characteristics.

Variable	Brain metastasis present (*N* = 154)	Brain metastasis absent (*N* = 2137)
Median age at diagnosis (range)	29 (16–52)	29 (13–75)
Primary site		
Testis	135 (88%)	1986 (93%)
Retroperitoneum	8 (5%)	82 (4%)
Mediastinum	10 (7%)	60 (3%)
Histology		
Seminoma	9 (6%)	449 (21%)
Non‐seminoma	147 (94%)	1742 (79%)
Choriocarcinoma	59 (38%)	89 (4%)
Embryonal carcinoma	32 (21%)	674 (32%)
Yolk sac tumor	12 (8%)	160 (8%)
Teratoma	12 (8%)	227 (11%)
Mixed	29 (19%)	513 (24%)
Metastatic sites		
Retroperitoneal LN	114 (74%)	1806 (85%)
Pulmonary	143 (93%)	855 (40%)
< 3 cm	66 (42.9%)	620 (29%)
≥ 3 cm	77 (50%)	235 (11%)
Liver	57 (37%)	211 (10%)
Bone	12 (8%)	57 (3%)
Brain metastasis time		
Diagnosis	98 (63.6%)	0 (0%)
1st relapse	38 (24.7%)	0 (0%)
≥ 2nd relapse	18 (11.7%)	0 (0%)
Pre‐chemo AFP ≥ 1000 ng/mL	23 (20%)	342 (25%)
Pre‐chemo hCG ≥ 5000 mIU/mL	105 (80%)	337 (25%)
Platinum refractory^a^	65 (42.2%)	175 (8.2%)

^a^ Defined as progression within 4 weeks of first‐line cisplatin‐based combination chemotherapy.

The vast majority of patients with BM had testis cancer as the primary site of disease (88%) and had non‐seminoma histology (96%). Out of the 154, 98 (63.3%) were found to have BM at time of initial presentation, 38 (24.7%) at time of 1st relapse, 14 (19.1%) at time of ≥ 2 relapse, and 4 (2.8%) had data missing. Most patients with BM had either pulmonary (93%) or retroperitoneal lymph node metastasis (74%). Among patients with BM, 64 (42%) received radiation only (whole‐brain radiotherapy or gamma knife), 22 (14%) had BM‐surgery only, 14 (9%) had both radiation and BM‐surgery, and 54 (35%) did not receive local therapy.

### 3.1 Survival

**FIGURE 1:**
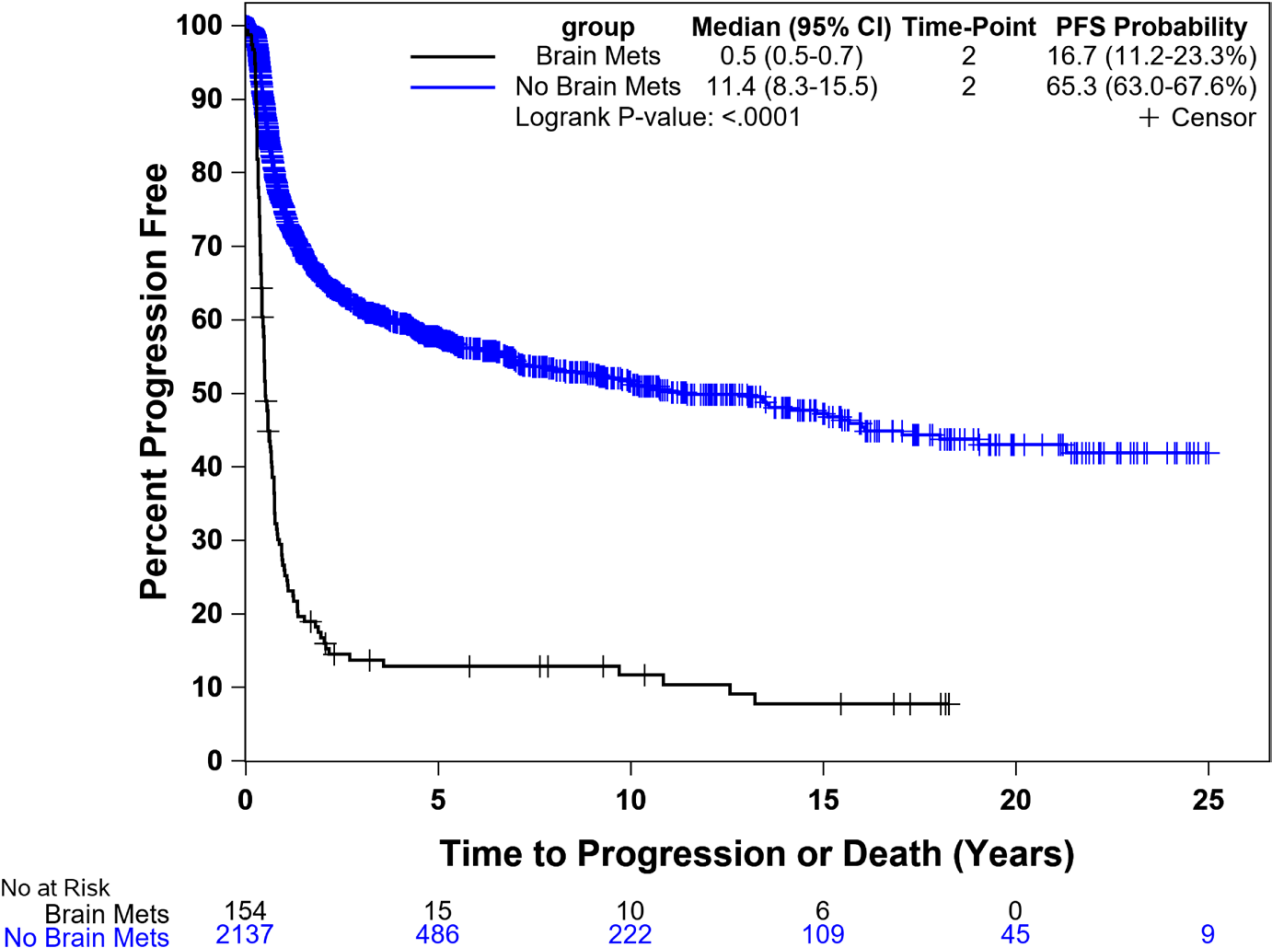
Progression‐free survival for patients with and without brain metastasis (BM).

**FIGURE 2:**
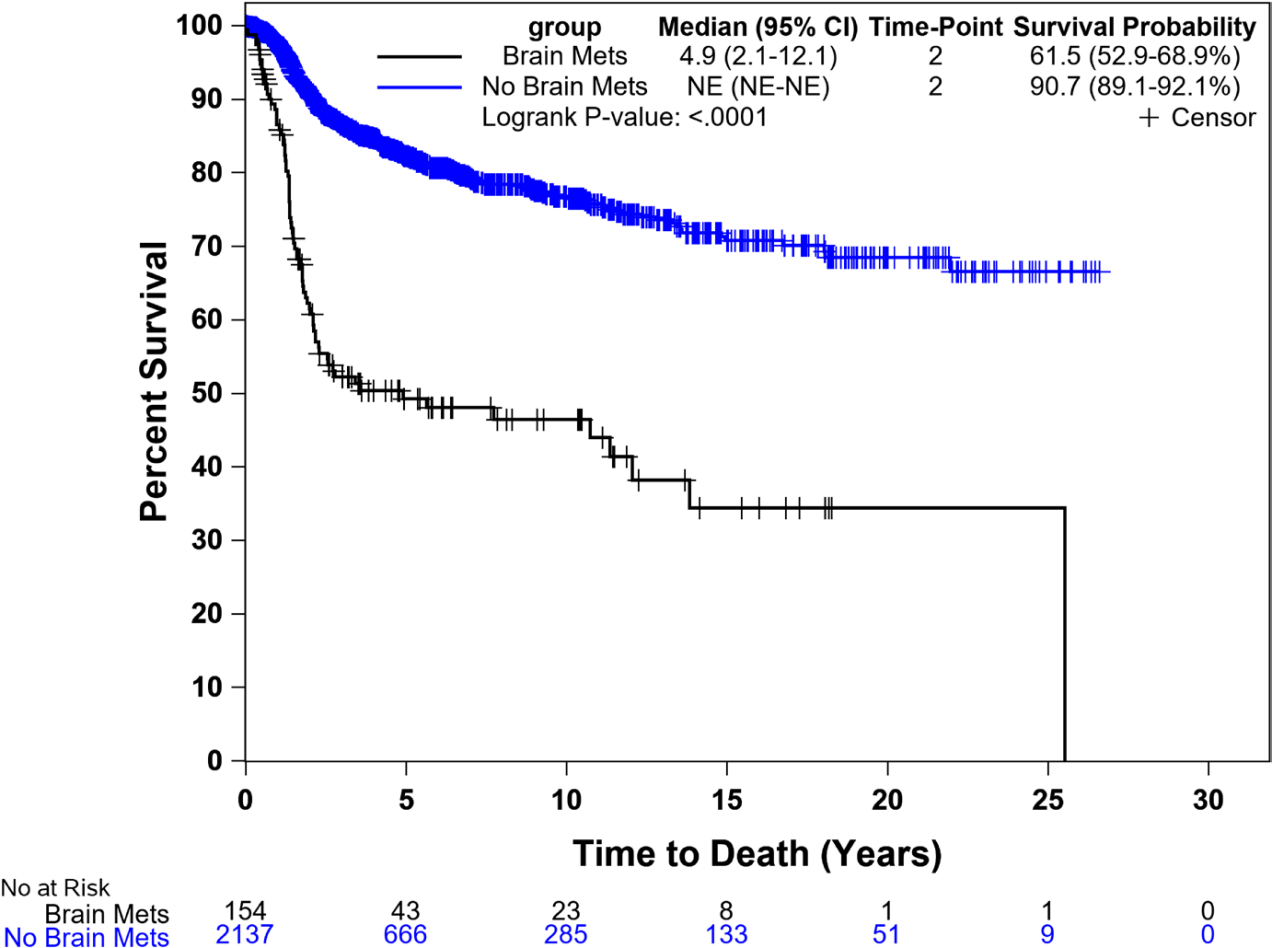
Overall survival (OS) for patients with and without brain metastasis (BM).

**FIGURE 3:**
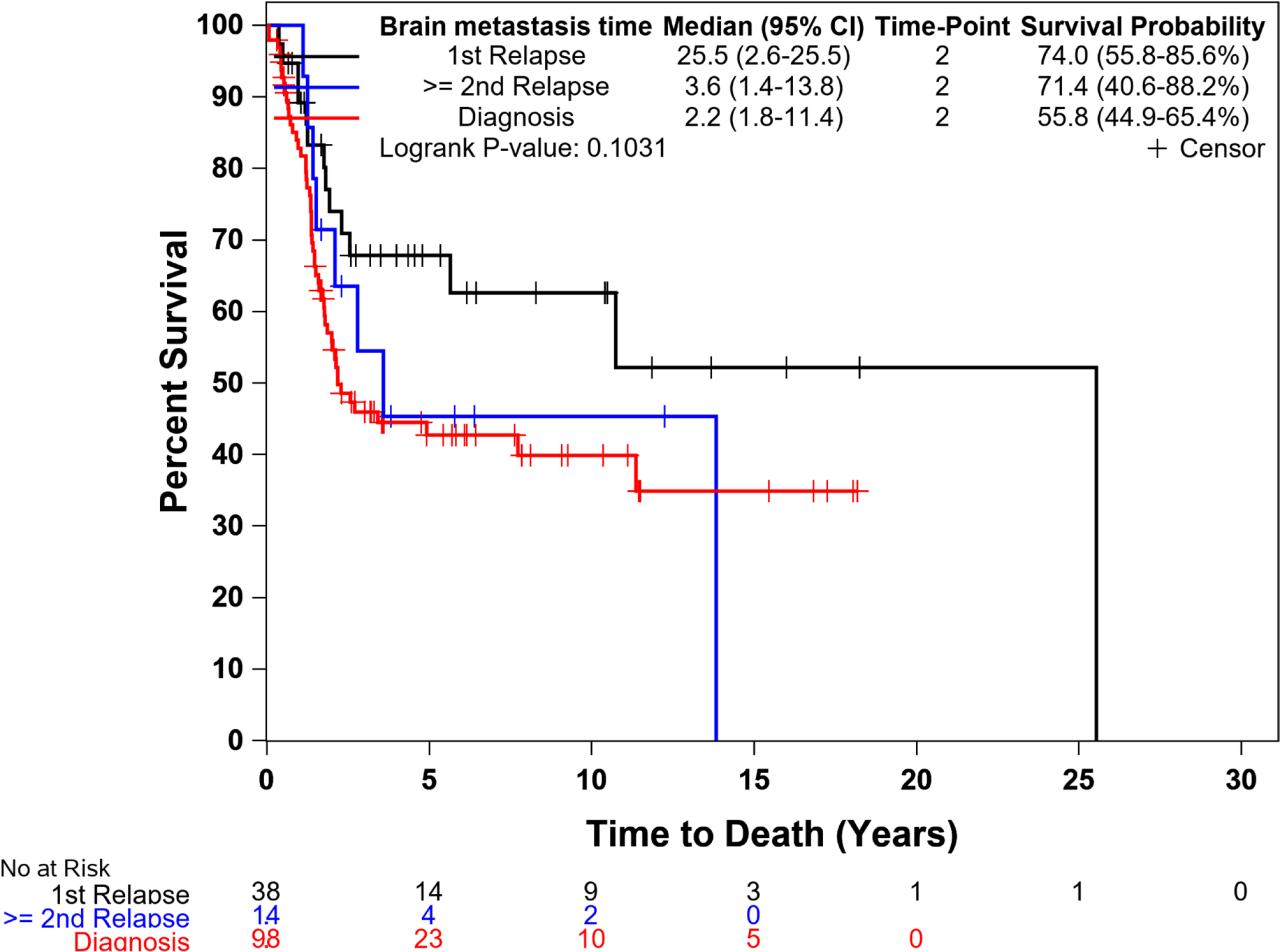
Overall survival (OS) for patients with brain metastasis (BM) at diagnosis, first relapse, and second relapse.

The 2‐year PFS for patients with BM was significantly lower compared to patients without BM (17% vs. 65%, *p* < 0.001) (Figure 1). The 2‐year OS for patients with BM was also significantly lower compared to patients without BM (62% vs. 91%, *p* < 0.001) (Figure 2). The median OS for patients who presented with BM at initial diagnosis was 2.2 years (95% CI, 1.8–11.4) compared to 25.5 (95% CI, 2.6–25.5) for subjects who had BM at time of 1st relapse and 3.6 (1.4–13.8) for those who had BM at 2nd relapse (Figure 3). Using the Cox proportional model for OS with diagnosis as the reference group, BM at time of 1st relapse was significantly different (HR: 0.52 (0.28–0.96), *p* = 0.036), but BM at time of 2nd relapse was not significantly different (HR: 0.86 (0.41–1.81), *p* = 0.690).

### 3.2 The Prediction Model

The model was constructed using a stepwise logistic regression model to determine the best model to move forward. The model with the highest ROC AUC included: age, histology, hCG value pre‐chemotherapy, presence of bone metastasis, and pulmonary metastasis size. Using these variables, the ROC AUC was 0.88 (*N* = 649). The model was tested on the validation dataset with a ROC AUC of 0.89 (*N* = 736). Using the beta values from the model when applied on the validation dataset, the closest integer was selected as the points to be assigned. The beta values for each variable and corresponding score are listed in Table 2. A total score was generated and run on the validation dataset to determine probability estimates for each possible total score value. Scores can range from 0 to 8, with higher scores predicting higher probability of BM.

**TABLE 2:** Point allocation for model predicting brain metastasis.

Risk factor	Categories	*β* Value	Points
Age	< 40 (reference)		0
	≥ 40	0.0785	1
Bone metastasis	No (reference)		0
	Yes	0.8836	1
Pulmonary metastasis size	None (reference)		0
	< 3 cm	1.9626	2
	≥ 3 cm	2.7559	3
hCG (mIU/mL)	< 5000 (reference)		0
	≥ 5000	1.2454	1
Histology	Non‐choriocarcinoma (reference)		0
	Choriocarcinoma	1.6776	2

**FIGURE 4:**
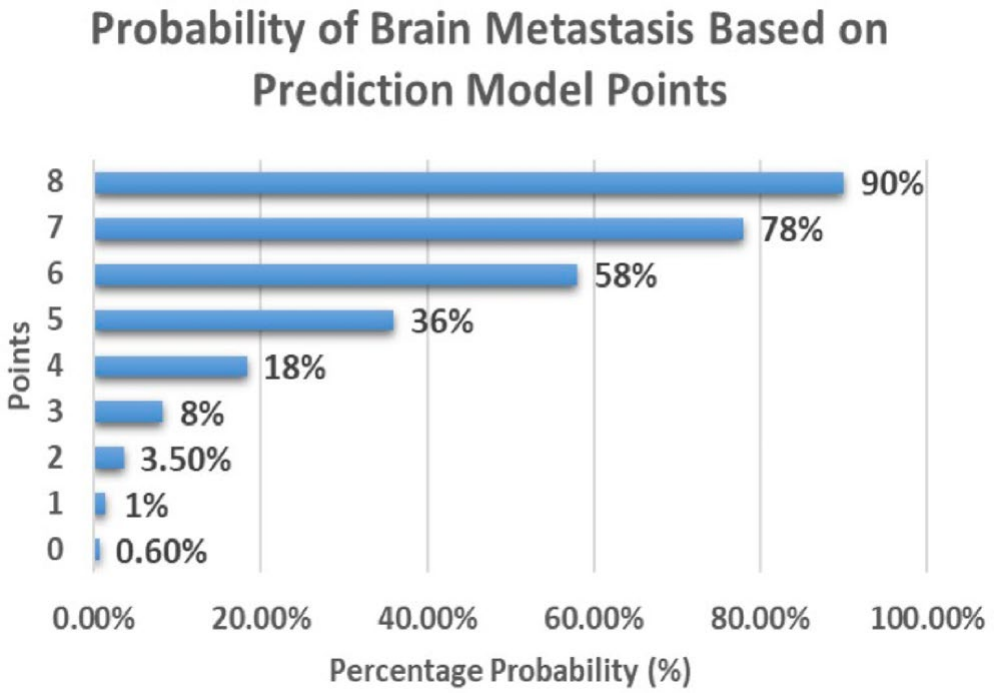
Prediction model score and probability of having brain metastasis (BM).

Patients with score of 0, 1, 2, 3, 4, 5, 6, 7, 8 points had a 0.6%, 1.4%, 3.5%, 8.2%, 18.3%, 36%, 58%, 78%, 90% probability of having BM, respectively (Figure 4).

## 4 Discussion

To the best of our knowledge, this is the first practical BM prediction model in patients with metastatic GCTs. The model was tested in the validation group to ensure reasonable accuracy. The model is simple, easy to use, and utilizes clinical variables that are commonly collected for all patients with metastatic GCTs. The size of pulmonary metastasis had the highest impact on the score and the probability of having BM. Choriocarcinoma histology was also associated with a high impact on the score and probability of developing BM. Patients with high scores (e.g., 6–8 points) have a 58%–90% probability of BM and may benefit from enhanced surveillance, including early brain imaging and multidisciplinary management strategies. Such targeted approaches could facilitate early BM detection, timely intervention, and potentially improved outcomes.

Our study showed a significant difference in PFS and OS in patients with and without BM, which is consistent with other studies [6]. In our study, more than 50% of patients with BM were dead in 5 years compared to less than 20% in patients without BM, which highlights the prognostic impact of BM.

In our study, patients who had BM at time of 1st relapse had significantly better OS compared to those who had it at time of initial presentation, which is not consistent with other studies that evaluated this before [6, 9]. Possible explanations include the common use of high dose chemotherapy in the relapsed setting at our center which is associated with improved outcomes [10]. There was no significant difference in outcomes between patients who had BM at 2nd relapse compared with patients who had it at time of initial presentation.

### 4.1 Limitations

This study has several limitations, including its retrospective design, selection bias, and missing data. Furthermore, the study was conducted at a single institution, which may limit the generalizability of the findings. Despite these limitations, the model demonstrates good discrimination in predicting BM occurrence in metastatic GCT patients.

## 5 Conclusion

This study proposes a predictive model for BM in metastatic GCTs that is both simple and reliable. The model can be utilized in clinical practice to identify high‐risk patients and guide early interventions. Further validation across larger and more diverse cohorts is needed.

## Author Contributions

**Tareq Salous:** writing – original draft (lead), writing – review and editing (equal). **Ryan Ashkar:** data curation (lead). **Sandra K. Althouse:** formal analysis (lead), writing – review and editing (equal). **Clint Cary:** writing – review and editing (equal). **Timothy Masterson:** writing – review and editing (equal). **Nasser H. Hanna:** writing – review and editing (equal). **Jennifer King:** writing – review and editing (equal). **Lawrence H. Einhorn:** writing – review and editing (equal). **Nabil Adra:** supervision (equal), writing – original draft (equal), writing – review and editing (equal).

## Ethics Statement

The study was approved by Indiana University Institutional Review Board (IRB). A waiver for consent is granted by the Indiana University IRB committee.

## Conflicts of Interest

The authors declare no conflicts of interest.

## Precis

The prediction model developed in this study demonstrated discrimination capability of predicting brain metastasis (BM) occurrence in mGCT and can be used to identify high‐risk patients.

## Data Availability

The data that support the findings of this study are available from the corresponding author upon reasonable request.
